# Maternal metformin treatment in obese pregnant dams normalizes neonatal sensory-motor reflexes

**DOI:** 10.1186/s40348-026-00259-8

**Published:** 2026-09-17

**Authors:** Elena Müller-Limberger, Bettina Frederick, Sebastian Hansen, Maria Wohlfarth, Ruth Janoschek, Esther Mahabir, Pascal Fischer, Dirk Gründemann, Simone Kalis, Miguel A. Alejandre Alcázar, Jörg Dötsch, Eva Hucklenbruch-Rother, Sarah Appel

**Affiliations:** 1 Department of Pediatrics and Adolescent Medicine, Faculty of Medicine and University Hospital Cologne, University of Cologne, Cologne, Germany; 2 Comparative Medicine, Center for Molecular Medicine Cologne (CMMC), Faculty of Medicine and University Hospital Cologne, University of Cologne, Cologne, Germany; 3 Department of Pharmacology, Faculty of Medicine and University Hospital Cologne, University of Cologne, Cologne, Germany; 4 Cologne Excellence Cluster on Cellular Stress Responses in Aging-Associated Diseases (CECAD), Faculty of Medicine and University Hospital Cologne, University of Cologne, Cologne, Germany; 5 Institute for Lung Health (ILH), University of Giessen and Marburg Lung Center (UGMLC) and Cardiopulmonary Institute (CPI), Member of the German Center for Lung Research (DZL), Giessen, Germany

**Keywords:** Maternal obesity, Metformin, Perinatal programming, Neonatal reflex ontogeny, Nervous system maturation, Developmental Origins of Health and Disease (DOHaD) concept

## Abstract

**Background:**

Maternal overweight and obesity have reached epidemic proportions over recent decades, with detrimental effects on offspring metabolism and neurodevelopment. Metformin, commonly used to treat type 2 diabetes mellitus and gestational diabetes, is being investigated for use in maternal obesity due to its weight-loss-promoting, glucose-lowering, and insulin-sensitizing effects. Metformin may improve maternal metabolic state and support early sensory-motor development in offspring.

**Methods:**

Three-week-old female C57BL/6NCrl mice were fed either a standard diet (SD, 9 kJ% fat, low sucrose content; CO group) or a Western-style diet (WSD, 45 kJ% fat, high sucrose content; OB group) from weaning through mating, gestation, and lactation. A subgroup of pregnant WSD-fed mice received metformin hydrochloride (target dose 380 mg/kg/d; OB + M group), dissolved in drinking water during gestation. At mid-gestation, a small cohort of pregnant dams was assessed for their metabolic profile. After delivery, pups were culled to six per litter at postnatal day (P) 2, and reflex ontogeny – such as righting, negative geotactic, and cliff-avoidance responses – was evaluated until P9 as a measure of early neurodevelopment.

**Results:**

Feeding a WSD resulted in approximately 20% heavier dams in the OB group by the time of mating. Metformin administration led to gestational weight gain of about 56%, compared to OB weight gain of approximately 64%. Plasma leptin levels, perigonadal white adipose tissue, fasting glucose, and HbA1c in OB + M dams were numerically intermediate between OB and CO at mid-gestation, without reaching statistical significance, while glucose tolerance remained impaired to a similar extent as in OB dams. Metformin reduced placental weight in female fetuses and increased the fetal-to-placental weight ratio compared with the CO group. Metformin had no effect on neonatal survival but accelerated postnatal weight gain in both sexes and improved sensory-motor reflex maturation compared to OB neonates, most consistently for the geotactic reflex in both sexes and the cliff avoidance reflex in females.

**Conclusions:**

Metformin did not restore maternal metabolic parameters at mid-gestation, despite the numerically intermediate phenotype across several markers, but it significantly attenuated gestational weight gain. Although neonatal survival was not improved, gestational metformin was associated with accelerated postnatal weight gain and improved sensory-motor reflex maturation in offspring, though effects varied across reflex types. Accelerated postnatal weight gain warrants cautious interpretation and long-term follow-up, as it is a risk factor for childhood obesity and metabolic disorders in later life.

**Supplementary Information:**

The online version contains supplementary material available at https://doi.org/10.1186/s40348-026-00259-8.

## Background

In recent years, the prevalence of maternal overweight and obesity has increased significantly worldwide, now representing a major public health concern. According to a recent meta-analysis, nearly half of all pregnancies globally involve maternal overweight, and approximately one in six pregnancies is complicated by maternal obesity [1]. Maternal obesity and related complications, such as gestational diabetes mellitus (GDM) or hypertension, are key factors influencing neonatal health, with higher incidences of macrosomia, neonatal intensive care admissions, and intrauterine death [2–7]. Beyond the perinatal period, an unfavorable intrauterine environment has been associated with adverse long-term effects on offspring, including a greater risk of metabolic and cardiovascular diseases later in life [8–10].

The concept of ‘Developmental Origins of Health and Disease’ (DOHaD) explains how adverse influences during the critical period of early development can permanently alter disease risk later in life. Initially described in adults born with low birth weight who later develop cardiovascular and metabolic disorders [11, 12, 13], it has since been broadened to encompass a wide range of chronic conditions, including disorders of central nervous system (CNS) development and function. Recent studies have specifically linked maternal obesity to impairments in cognitive performance, emotional regulation, and behavioral outcomes in children and adolescents [14–19]. According to the DOHaD hypothesis, the placenta acts as a crucial mediator translating maternal metabolic stress into altered fetal brain programming [20].

Assessing sensory-motor reflexes in the neonatal period provides an early and sensitive indicator of CNS maturation. In both humans and rodents, delays in the acquisition of primitive reflexes such as righting, grasping, and postural responses have been associated with adverse prenatal conditions and an increased risk of later neurodevelopmental disorders [21–24]. In murine CNS development, cortical neurogenesis begins at mid-gestation and is highly sensitive to maternal inflammatory and metabolic perturbations [25]. The cerebellar and vestibular circuits that underlie neonatal sensory-motor reflexes are simultaneously being established at mid-gestation, with cerebellar GABAergic neurons at gestational days (G) 10–13 [26] and vestibular ganglion cells born at G10–14 [27]. In experimental models, maternal high-fat diet (HFD) consistently delays physical development and impairs reflex ontogeny in offspring [28–30], suggesting disrupted sensory-motor integration or delayed CNS maturation.

Given the far-reaching implications of maternal obesity for offspring metabolic and neurodevelopmental health, strategies to mitigate adverse intrauterine programming are urgently needed. Metformin, an orally administered dimethyl biguanide used to treat type 2 diabetes mellitus (T2DM) and GDM, is under investigation as a treatment for maternal obesity because of its weight-loss-promoting, glucose-lowering, and insulin-sensitizing effects [31]. In pregnant women with obesity, metformin has been shown to reduce gestational weight gain, decrease the risk of pre-eclampsia and cesarean delivery [32–35]. Notably, metformin crosses the placenta and can reach fetal concentrations comparable to maternal levels [36], raising concerns regarding its programming effects on offspring development. While maternal benefits are well documented and no association with teratogenic or congenital fetal outcomes has been described [37], its long-term impact on fetal and neonatal CNS development remains unclear.

In this study, we investigated whether maternal metformin treatment during pregnancy in obese dams improves maternal metabolic outcomes and protects offspring neurodevelopment. Using a well-established diet-induced obesity (DIO) mouse model, we assessed maternal and fetal pregnancy-related outcomes, as well as early postnatal development of sensory-motor reflexes as indicators of neurodevelopmental maturation. We collected maternal and fetal tissues at G13.5, a critical mid-gestational timepoint when labyrinth transport becomes an absolute requirement for fetal nutrient supply [38], and when the maternal obese environment most directly impacts placental function as a DOHaD mediator. Our findings offer new insights into the programming effects of metformin in the context of maternal obesity and contribute to the ongoing debate regarding its clinical use as a prenatal intervention during obese pregnancy.

## Methods

### Animals, husbandry, and diets

**Fig. 1:**
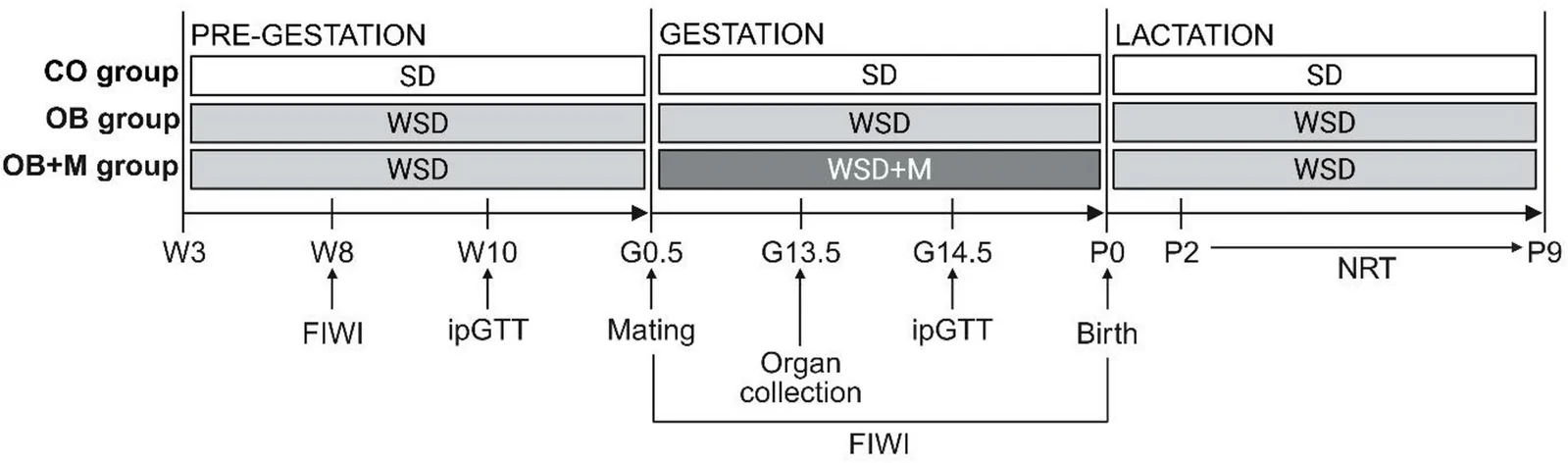
Study design. Young female C57BL/6NCrl mice were assigned to control (CO), obese (OB), or obese with gestational metformin treatment (OB + M) groups and fed either a standard diet (SD) or a Western-style diet (WSD) during pre-gestation, gestation, and lactation. Feed and water intake (FIWI) were measured in eight-week-old (W8) mice during pre-gestation or later throughout gestation. An intraperitoneal glucose tolerance test (ipGTT) was performed pre-gestation in W10 mice and during gestation at G14.5. After delivery, the number of neonates was reduced to six pups per litter at P2. Offspring were assessed for fetal characteristics at G13.5 or for neonatal characteristics at P2. Early postnatal development was examined using neonatal reflex tests (NRT) from P2 to P9.

This study followed the Animal Research Reporting of In Vivo Experiments (ARRIVE) guidelines, version 2.0 [39]. All animal handling and procedures complied with German regulations and legal requirements for animal welfare and were approved by the relevant authorities. The experimental design, housing conditions, dietary interventions, and breeding strategy are described in detail elsewhere [40]. In brief, weaned C57BL/6NCrl mice obtained from Charles River Laboratories (Sulzfeld, Germany) were housed in small groups of three under standard conditions (room temperature (RT) 22 ± 2 °C, humidity 50–60%, and a 12/12 h light/dark cycle starting at 6.00 a.m.) in individually ventilated cages (type II long, blue line by Tecniplast, Buguggiate, Italy) at the animal facility of the Medical Faculty, University of Cologne, with *ad libitum* access to food and water. Female mice received either a standard diet (SD; V1534-000, Ssniff, Soest, Germany) containing 9 kJ% fat and 4.7% sucrose or a Western-style diet (WSD; E15744-344 (DIO), Ssniff) containing 45 kJ% fat and 21% sucrose from weaning throughout pregnancy and lactation (for detailed information on dietary content, see Add. Table 1). After mating at 12–16 weeks of age, a subset of WSD-fed dams was treated with metformin hydrochloride (CAS 1115-70-4, LKT Laboratories, St. Paul, MN, USA) at a target dose of 380 mg/kg per body weight per day (mg/kg/d) dissolved in drinking water until offspring were born (Fig. 1). For the mid-gestation cohort (G13.5), mating was performed overnight for one night only, with males removed the following morning. The morning after the mating night was designated as G0.5, allowing precise and consistent determination of gestational age across all animals. For the cohort that carried pregnancies to term, mating was performed over two consecutive nights for the CO and OB group, while dams of the OB + M group were mated for one night only. Postnatal assessments for CO and OB were therefore initiated at a fixed postnatal timepoint (postnatal day (P) 1) rather than relative to a precisely determined gestational age. After delivery, animal caretakers counted the pups on P1. On P2, litters were reduced to six pups each, with a balanced sex ratio, if applicable. Dams with fewer than five offspring were excluded from the study, unless litter size could be supplemented to at least five pups by cross-fostering of surplus pups from a litter of the same experimental group.

### Feed and water intake in dams before and during pregnancy

The feed and water intake (FIWI) was measured to evaluate and compare consumption, as previously detailed [40]. The FIWI was studied in small groups (2–3 cage mates) of female mice aged eight weeks before pregnancy for 10 consecutive days. Group housing was maintained during this period in accordance with animal welfare guidelines, as individual housing represents a significant stressor for mice; FIWI was therefore measured at the cage level and divided by the number of animals to estimate individual consumption. During pregnancy, dams were housed individually. Consumption was determined by regularly weighing the setup (three times per week) and recording the difference from the previous measurement. The average daily consumption per animal was calculated for each period.

### Intraperitoneal glucose tolerance test in dams before and during pregnancy

The intraperitoneal glucose tolerance test (ipGTT) was performed in female mice either before pregnancy at 10 weeks of age or in pregnant dams on G14.5, as previously described [40]. Briefly, at the start of the light cycle, following the mice’s active (dark) phase, a baseline non-fasting blood glucose level was measured from a tail-tip blood drop using a conventional blood glucose meter (GlucoNavii, IMACO GmbH, Lüdersdorf, Germany). Mice were then fasted for six hours during the inactive (light) phase, and glucose tolerance was assessed by administering a single intraperitoneal injection of 20% glucose solution (B. Braun, Melsungen, Germany) at a dose of 2 g/kg per body weight. Blood glucose levels were measured before glucose administration (0 min) and at 15, 30, 60, and 120 min post-injection.

### Neonatal reflex tests

Neonatal reflex tests (NRT) were used to assess the early development of sensory-motor skills and were conducted daily at the start of the dark/active phase, between 7:00 and 9:00 p.m., in newborn pups from P2–P9. All three reflexes are mediated by vestibular, proprioceptive, and vibrissae sensory inputs rather than visual input; testing was therefore deliberately conducted within the pre-visual developmental window (P2–P9), prior to eyelid opening (P10–P14), to specifically assess the maturation of these non-visual sensory-motor circuits.

One male and one female pup per litter were randomly assigned to NRT and marked daily with a waterproof cosmetic pen to ensure that the same animal was tested. Proper weight gain (0.3 ± 0.2 g/day) and the presence of a milk spot were monitored daily to detect any experimental stress. Each test lasted up to 30 s and was performed twice with an inter-trial interval of 30 s. The proper development of sensory-motor skills was indicated by a decrease in the time required for each trial. Reflex tests were conducted sequentially, starting with the righting reflex and ending with the cliff avoidance reflex.

#### Righting reflex

The righting reflex (RR) assesses the neonate’s ability to turn from the dorsal to the ventral position. The test was considered successful when the animal could rotate onto all four paws within 30 s. If the pup could not turn into the ventral position within 30 s, the test was stopped and scored as the maximum duration (30 s).

#### Geotactic reflex

The geotactic reflex (GR) assesses the neonate’s ability to rotate upwards on a downward inclined surface. For this, the neonate was placed with its nose tip facing down on a Plexiglas plate tilted by 30°. The test was considered successful when the animal turned 180° upward within 30 s. If the pup did not turn 180° upwards, the test was scored as the maximum duration.

#### Cliff avoidance reflex

The cliff avoidance (CA) reflex assesses the neonate’s ability to turn away from an abyss. For this, the neonate was placed on an elevated platform with its nose tip and front paws elevated. The test was considered successful when the animal turned its body by 90° away from the edge within 30 s. If the pup was falling or did not turn away by 90°, the test was scored as the maximum duration.

The area under the latency time curve (AUC) was calculated with GraphPad Prism (version 10.1.2, La Jolla, CA, USA) across P2–P9 for every individual animal, providing an integrative measure of the cumulative latency burden over the neonatal observation period, analogous to the AUC approach applied to ipGTT data. To cross-validate the AUC results, we additionally determined the ‘day of criterion’ for each group, defined as the first postnatal day on which the group median latency fell at or below an empirically derived threshold for mature reflex performance. Thresholds were derived from control group (CO) data: ≤ 5 s for the RR and CA (reflecting stable late-window CO medians for these reflexes), and ≤ 8 s for the GR (reflecting the characteristically higher baseline latencies of this reflex in the P2–P9 window).

### Euthanasia and tissue preparation

To investigate characteristics related to mid-pregnancy, pregnant dams and their fetuses were examined at G13.5. Pregnant dams were euthanized by CO_2_ inhalation after a four-hour fast to ensure comparable metabolic conditions. Body weight was recorded, and blood samples were collected via cardiac puncture into lithium heparin tubes (Microvette, Sarstedt, Nürmbrecht, Germany) to measure plasma metformin concentrations and enable subsequent metabolic biomarker analyses. Surplus blood was used to assess glycated hemoglobin (HbA1c) levels. Perigonadal white adipose tissue (pgWAT) was weighed in pregnant dams. The uterus containing the amniotic sacs with fetuses was removed, and individual fetal-placental units were further examined. A resorption rate was calculated by counting all implantation sites and comparing the number of live fetuses with the number of resorbed fetuses. All live fetuses from each dam were weighed, and placentas were weighed only for fetuses exceeding the standardized litter size of six (i.e., placentas 7–11), due to tissue allocation for other experiments. Fetuses were decapitated, tail tip cuts were collected for sex determination, and whole fetuses were stored at -80 °C for metformin concentration measurement.

### Determination of blood parameters

#### Glycated hemoglobin analysis from whole blood samples

HbA1c was measured in pregnant dams at G13.5 using the DCA Vantage Analyzer with the corresponding HbA1c Reagent Test Kits (Siemens Healthineers, Erlangen, Germany). The HbA1c concentration was determined following the manufacturer’s protocol. Briefly, test cartridges were brought to RT before testing. Then, the capillary holder device was filled with fresh blood samples obtained by cardiac puncture.

#### Plasma sample preparation

Fresh blood samples from pregnant dams at G13.5 collected into lithium heparin tubes were centrifuged at RT for 5 min at 2,000 × g to separate plasma. The upper plasma layer was aliquoted into reaction tubes and stored at -80 °C until further analysis.

#### Metabolic biomarkers analysis

Plasma samples from pregnant dams at G13.5 were analyzed as previously described [40]. Plasma was examined for adiponectin (MADPNMAG-70 K-01, RRID: AB_2783857, MILLIPLEX, Merck Millipore, Darmstadt, Germany), insulin, leptin, resistin, monocyte chemoattractant protein 1 (MCP-1), interleukin-6 (IL-6), and tumor necrosis factor alpha (TNFα) levels (MADKMAG-71 K, RRID: AB_2801416, MILLIPLEX) using Multiplex Enzyme-Linked Immunosorbent Assay (ELISA). Procedures followed the manufacturer’s protocols, with analyses performed on Bio-Plex 200 Systems (Bio-Rad Laboratories, Hercules, CA, USA) using Bio-Plex Manager software (version 6.1, Bio-Rad Laboratories). Absolute cytokine concentrations were determined by comparing the median fluorescence intensity of the samples to the standard curves. Samples were measured in duplicates with a coefficient of variation ≤ 15% between duplicates. The measured concentrations include interpolated data. The adiponectin-to-leptin ratio (A/L) was calculated according to Frühbeck et al. (2017) [41] as a measure of adipose tissue dysregulation.

#### Determination of metformin concentration from plasma samples and whole fetal lysates

Metformin levels in plasma samples and whole fetal lysates were measured using an established liquid chromatography-mass spectrometry (LC-MS) method [42, 43]. Samples were pre-processed as follows: to determine metformin concentration in maternal plasma, samples were diluted 1:100 with acetonitrile (ACN). For the determination of metformin concentration in whole fetuses, samples were mixed with 300 µL of 4 mM perchloric acid (HClO_4_) and homogenized using a Precellys homogenizer (P000918-LYSK0A, Bertin Technologies, Paris, France) at medium speed for 2 × 10 s. Homogenates were briefly centrifuged at 16,000 × g at 4 °C, and 100 µL of the resulting supernatant was transferred to a new tube and precipitated with 400 µL of ice-cold (-20 °C) ACN. Samples were vortexed and centrifuged at 16,000 × g for 20 min at 4 °C. The supernatant was stored at -80 °C until further analysis. Before LC-MS measurement, fetal lysate samples were diluted 1:10 with ACN.

The following fragments were selected for reaction monitoring (m/z parent, m/z fragment, collision energy (V)): metformin 130, 71, 29 V and proline 116, 70, 22 V. For each analyte, the peak area was obtained by integrating the intensity above background vs. time in the appropriate elution interval. Proline was chosen to normalize the LC-MS data. To calculate the metformin concentration in fetuses, after normalizing to proline, the concentration was determined relative to fetal body weight and averaged by two fetuses per dam.

### Sex determination

#### Isolation of genomic DNA from tail tips

Sex determination was conducted as previously described [44]. Briefly, frozen tail tips of fetuses or placenta fragments were lysed using lysis buffer (100 mM Tris, 5 mM EDTA, 200 mM NaCl, and 0.2% SDS at a pH of 8.0) supplemented with 0.01% Proteinase K (Thermo Fisher Scientific, Waltham, MA, USA) overnight at 55 °C and 300 rpm. Genomic DNA (gDNA) was precipitated by mixing the samples with 100% isopropanol and incubating them at RT for 10 min. Samples were centrifuged for 15 min at 16,000 × g at 4 °C. The supernatant was discarded, and the remaining pellet was washed with ice-cold 70% ethanol. The pellet was then centrifuged again, and the supernatant was discarded. The remaining gDNA pellet was air-dried and then dissolved in ultra-pure HPLC-grade H_2_O. Samples were frozen at -20 °C until the polymerase chain reaction (PCR) was performed.

#### Genotyping polymerase chain reaction and agarose gel electrophoresis

The PCR was carried out as previously described [44]. Tail tip or placental fragment genomic DNA (gDNA) served as the template for the genotyping PCR. For this, 1 µL of gDNA was mixed with 9 µL of PCR mix (1× GoTaq Flexi buffer, Promega, Madison, WI, USA), 3 mM MgCl₂, 0.2 mM dNTPs, 0.4 µM of each primer, and 0.5 units of GoTaq (Promega) to amplify fragments of the X (104 bp) and Y (344 bp) chromosomes. Samples were amplified using a PCR cycler (Biometra TOne, Analytik Jena GmbH, Jena, Germany) following this protocol: initial denaturation at 95 °C for 3 min, then 35 cycles of 95 °C for 30 s, 58 °C for 1 min, and 72 °C for 1 min, with a final elongation step at 72 °C for 10 min. For the X chromosome, the primer pair X-for 5´-AACATCCTGAACACCTTGCC-3´ and X-rev 5´-TAGCTTGTGGCTCTCCAGGT-3´ was used. For the Y chromosome, the primer pair Y-for 5´-CTGGAGCTCTACAGTGATGA-3´ and Y-rev 5´-CAGTTACCAATCAACACATCA-3´ was used. PCR products were loaded onto a 1.5% (w/v) agarose gel (1× TAE buffer) supplemented with 0.0001% Midori Green (Nippon Genetics Europe GmbH, Düren, Germany). Electrophoresis was performed for 30 min at 110 V and 200 mA. The PCR products were visualized using an UV chamber (BioDocAnalyze, Biometra, Analytik Jena). The number and size of the bands indicated whether the samples were male (two bands at 104 bp and 344 bp) or female (one band at 104 bp).

### Statistics

Data were analyzed and visualized using GraphPad Prism (version 10.1.2) and are presented as scatterplots with bars or as line charts with error lines indicating mean ± standard error of the mean (SEM). The normality of the data was assessed using the D’Agostino and Pearson normality test. Pairwise comparison of normally distributed data was analyzed using Student’s t-test, Mann-Whitney test for non-normally distributed data, or Welch’s t-test for unequal variances. Comparison of more than two groups of normally distributed data with a sample size > 7 was analyzed using one-way ANOVA and Tukey’s post-hoc test, Kruskal-Wallis test and Dunn’s post-hoc test for non-normally distributed data or sample size < 7, or Welch’s ANOVA and Dunnett’s post-hoc test for unequal variances. Line charts were analyzed using repeated-measurements (RM) mixed-effect analysis and Bonferroni’s post-hoc test or two-way RM ANOVA and Tukey’s post-hoc test. Grouped analyses were performed using two-way RM ANOVA and Tukey’s post-hoc test. Detailed sample sizes and the statistical tests used are provided in the figure and table legends. In all analyses involving fetal or neonatal outcome measures, the dam was defined as the biological replicate. G13.5 body weight data are presented as litter means calculated from all live fetuses per dam (1–8 fetuses per litter). Placental weights at G13.5 were obtained only from a subset of placentas per litter (1–4 placentas per litter), as the majority were retained with the uterus, amniotic sac, and umbilical cord intact for allocation to histological and molecular analyses. Body weight and body length of neonates at P2 were measured exclusively in surplus pups culled during litter standardization; the number of pups available per litter therefore varied depending on litter size (1–4 pups per litter).

### AI use statement

During the preparation of the revised manuscript, the authors used Claude (Anthropic, version Sonnet 4.6) as an AI-assisted writing and analysis tool. Specifically, the tool was used to support literature searches and to assist in drafting and revising text passages. The tool was not used for data collection, primary statistical analysis, or generation of figures. All AI-assisted content was critically reviewed, verified, and revised by the authors, who take full responsibility for the accuracy and integrity of all content in this manuscript.

## Results

### Feeding a WSD robustly induced obesity and impaired glucose tolerance in pre-pregnant mice

**Fig. 2:**
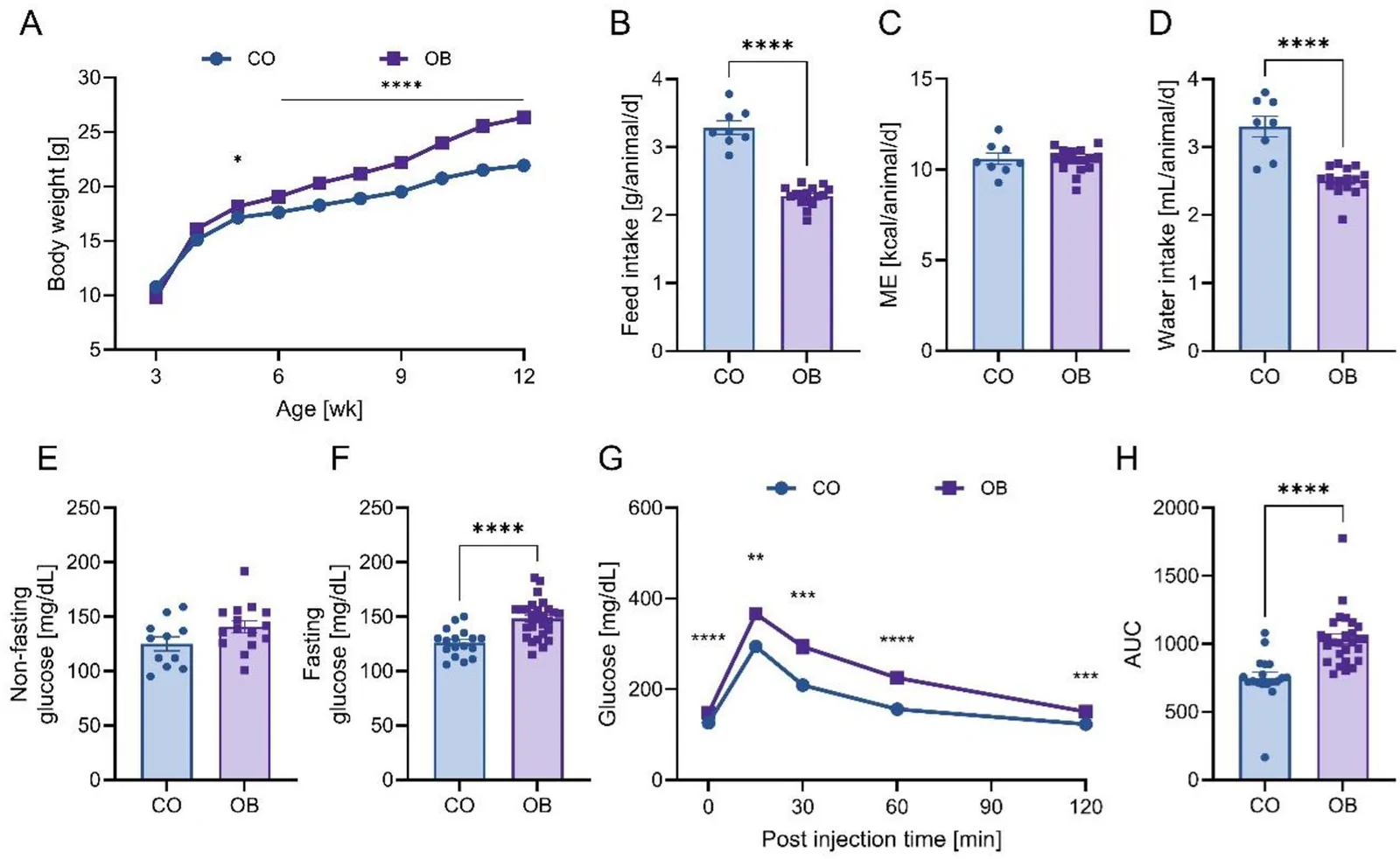
Pre-pregnancy-related data of female mice. **A** Body weight history from weaning until mating in control (CO; *n* = 26) and obese (OB; *n* = 99) female mouse groups. **B** Averaged mean feed intake, **C** mean metabolizable energy, and **D** mean water intake of eight-week-old female mice of the CO (*n* = 8) and OB (*n* = 16) groups. **E** Non-fasting glucose, **F** glucose after six hours of fasting, **G** glucose tolerance test, and **H** area under the curve (AUC) in ten-week-old female mice of the CO (*n* = 11–17) and OB (*n* = 15–27) groups. Data are presented as mean ± SEM, mixed-effects analysis + Bonferroni’s post-hoc test (**A**), unpaired Student’s t-test (**B** + **C** and **E** + **F**), Mann-Whitney test (**D)**, two-way repeated-measurements ANOVA + Bonferroni’s post-hoc test (**G**), and Welch’s t-test (**H**); * *p* < 0.05, ** *p* < 0.01, *** *p* < 0.001, **** *p* < 0.0001.

Young female mice were fed either a SD or a high-fat/high-sucrose WSD from three weeks of age until mating and throughout gestation and lactation (Fig. 1). By five weeks of age, the WSD-fed mice, referred to as the obese (OB) group, had already begun to differ in body weight from their SD-fed counterparts, referred to as the control (CO) group (Fig. 2A). By 12 weeks of age, the OB group was approximately 20% heavier than the CO group (26.32 ± 0.276 vs. 21.93 ± 0.341 [g]; *p* < 0.0001; Fig. 2A).

During the pre-gestation phase, feed and water intake were measured at approximately eight weeks of age, indicating that the increased weight gain observed in the OB group was not due to hyperphagia. Feed intake was significantly lower in the OB group compared to the CO group (2.277 ± 0.0370 vs. 3.286 ± 0.0987 [g/animal/d]; *p* < 0.0001; Fig. 2B); however, the conversion of the consumed SD or WSD into metabolizable energy showed the same caloric intake between the groups (Fig. 2C). Water intake was also reduced in the OB group compared to the CO group (2.504 ± 0.0502 vs. 3.301 ± 0.1507 [mL/animal/d]; *p* < 0.0001; Fig. 2D), likely due to higher overall feed consumption in the CO group.

The increased weight gain observed in the OB group was associated with impaired glucose tolerance, as measured in a cohort of CO and OB mice at 10 weeks of age. While non-fasting glucose values were similar between the groups (Fig. 2E), glucose levels were higher after a six-hour fasting period in the OB group compared to the CO group (148.2 ± 3.35 vs. 126.1 ± 3.06 [mg/dL]; *p* < 0.0001; Fig. 2F). During the intraperitoneal glucose tolerance test (ipGTT), the glucose levels of the OB group were significantly elevated at 15, 30, 60, and 120 min after glucose injection (Fig. 2G), and the area under the curve (AUC) was notably greater in the OB group compared to the CO group (1036.0 ± 38.5 vs. 750.6 ± 43.2; *p* < 0.0001; Fig. 2H), indicating impaired glucose tolerance.

### Maternal metformin treatment in obese pregnant dams reduced gestational weight gain independently of improved glucose tolerance

**Fig. 3:**
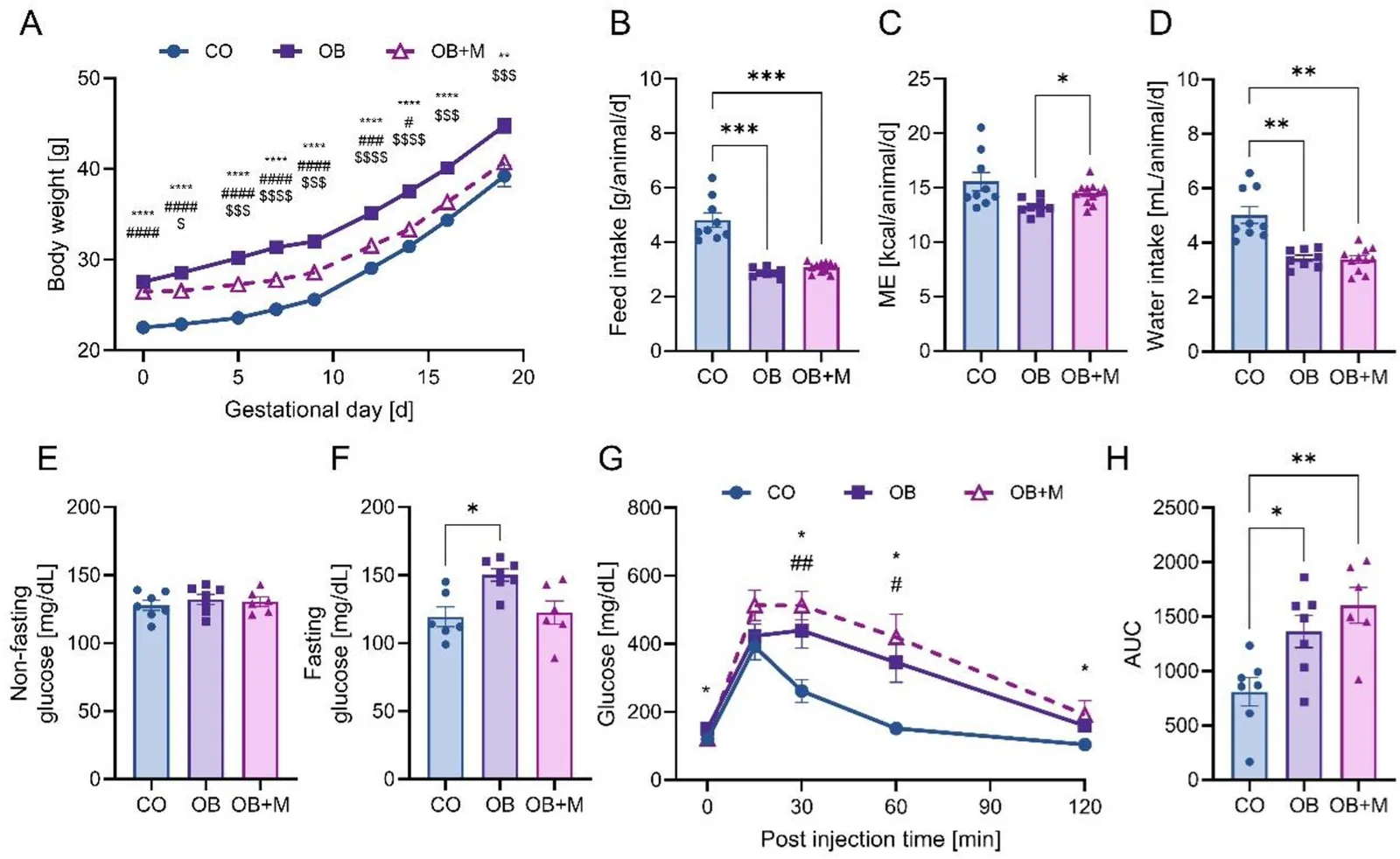
Pregnancy-related data of breeding dams. **A** Body weight history of pregnant dams in control (CO; *n* = 29), obese (OB; *n* = 39), and obese with gestational metformin treatment (OB + M; *n* = 35) groups. **B** Mean feed intake, **C** mean metabolizable energy (ME), and **D** mean water intake throughout gestation in pregnant dams of CO (*n* = 9), OB (*n* = 8), and OB + M (*n* = 11) groups. **E** Non-fasting glucose, **F** glucose after six hours of fasting, **G** intraperitoneal glucose tolerance test (ipGTT), and **H** area under the curve (AUC) at mid-gestation (G14.5) in pregnant dams of CO (*n* = 6–7), OB (*n* = 7), and OB + M (*n* = 6) groups. Asterisk (*) indicates differences between OB and CO, hash (^#^) indicates differences between OB + M and CO, and dollar (^$^) indicates differences between OB + M and OB (in line charts). Data are presented as mean ± SEM, mixed-effects analysis + Tukey’s post-hoc test (**A**), Welch’s ANOVA + Dunnett’s post-hoc test (**B**, **C**, **D** and **H**), Kruskal-Wallis + Dunn’s post-hoc test (**E** + **F**), and two-way repeated-measurements ANOVA + Tukey’s post-hoc test (**G**); */^#^/^$^*p* < 0.05, **/^##^/^$$^*p* < 0.01, ***/^###^/^$$$^*p* < 0.001, ****/^####^/ *p* < 0.0001.

At 12–16 weeks of age, CO and OB mice were mated overnight. After mating, a subgroup of the OB group was given metformin hydrochloride, referred to as the OB + M group. Metformin water intake resulted in an average dose of 433.2 ± 18.08 mg/kg/d throughout pregnancy (Add. Figure 1A + B), which was slightly higher than the target concentration of 380 mg/kg/d. Pregnant OB + M dams showed a significant reduced body weight after approximately three days of metformin administration compared to OB dams (26.567 ± 0.521 vs. 28.557 ± 0.499 [g]; *p* = 0.0207; Fig. 3A). The decreased weight gain in OB + M dams persisted over the rest of the gestation period, resulting in an overall reduced percentage weight gain at the end of the gestational period compared to CO dams (55.94 ± 3.43 vs. 69.99 ± 4.84 [%]; *p* = 0.0322), while there was no difference between OB + M and OB dams (Add. Figure 1C).

During pregnancy, feed and water intake were monitored and showed decreased feed consumption in both OB dams (2.881 ± 0.0583 vs. 4.814 ± 0.2642 [g/animal/d]; *p* = 0.0002) and OB + M dams (3.081 ± 0.0586 vs. 4.814 ± 0.2642 [g/animal/d]; *p* = 0.0004) compared to CO dams (Fig. 3B). Conversion of the SD and WSD into metabolizable energy revealed a reduced caloric intake in OB dams compared to OB + M dams (13.30 ± 0.269 vs. 14.51 ± 0.299 [kcal/animal/d]; *p* = 0.0225), while OB + M dams did not differ from CO dams (Fig. 3C). Water intake was lower in both OB dams (3.428 ± 0.1166 vs. 5.020 ± 0.3097 [mL/animal/d]; *p* = 0.0021) and OB + M dams (3.388 ± 0.1314 vs. 5.020 ± 0.3097 [mL/animal/d]; *p* = 0.0015) compared to CO dams (Fig. 3D), likely due to higher overall feed consumption in the CO group.

At G14.5, glucose tolerance was examined in a sub-cohort of pregnant dams. Non-fasting glucose levels did not differ between the groups (Fig. 3E). After a six-hour fast, OB dams displayed higher glucose levels compared to CO dams (150.1 ± 4.49 vs. 119.3 ± 7.24 [mg/dL]; *p* = 0.0132), while OB + M dams did not differ from either group (Fig. 3F). Despite elevated glucose levels observed in the ipGTT in the OB group compared to the CO group, the OB + M group, contrary to our expectations, also showed high glucose levels compared to the CO group. The OB group exhibited higher glucose levels at 30, 60, and 120 min after glucose injection compared to the CO group. The OB + M group differed from the CO group at 30 min and 60 min post-injection (Fig. 3G). Both the OB group (1364.0 ± 147.6 vs. 809.4 ± 128.3; *p* = 0.0347) and OB + M group (1603.0 ± 164.8 vs. 809.4 ± 128.3; *p* = 0.0040) showed increased AUC compared to the CO group (Fig. 3H). Therefore, although metformin reduced excessive gestational weight gain, it did not improve glucose tolerance in OB + M dams.

### Maternal metformin treatment in obese pregnant dams reduced maternal obesity related characteristics without improving neonatal survival

Another sub-cohort of pregnant dams was studied for their obesity-related traits during pregnancy at G13.5. Metabolic biomarkers associated with obesity (adiponectin, insulin, leptin, resistin, MCP-1, IL-6, and TNFα) were measured in plasma samples using multiplex ELISA and revealed higher leptin levels in OB dams compared to CO dams (Table 1), whereas OB + M dams did not (statistically) differ from CO dams. Other obesity-relevant hormones and chemokines (adiponectin, insulin, resistin, and MCP-1) showed no differences or were below the detection limits (IL-6: 3.07 pg/mL and TNFα: 3.04 pg/mL; not shown). The adiponectin-to-leptin (A/L) ratio was calculated as an indicator of adipose tissue dysregulation and was significantly lower in OB dams compared to CO dams (Table 1), while OB + M dams did not differ from either group. The HbA1c level was higher in OB dams than in CO dams (Table 1), with OB + M dams showing no difference from either group. Lastly, plasma metformin concentrations were measured in this cohort of OB + M dams, with an average concentration of 3.0 ± 0.39 µg/mL (*n* = 5).f

**Table 1:** Obesity-related parameters measured in plasma or whole blood samples of pregnant dams at mid-gestation (G13.5).

	CO	OB	OB + M	*p* value (Kruskal-Wallis)
**Plasma parameters**				
Adiponectin [µg/mL]	14.4 ± 2.0	13.4 ± 3.2	11.3 ± 4.3	ns
Insulin [pg/mL]	396.4 ± 80.5	673.8 ± 183.2	479.0 ± 191.5	ns
Leptin [pg/mL]	26.4 ± 6.3	723.0 ± 139.9**	270.9 ± 51.5	< 0.0001
Resistin [pg/mL]	1140.4 ± 162.8	991.3 ± 187.5	915.0 ± 160.9	ns
MCP-1 [pg/mL]	4.9 ± 2.0	11.8 ± 7.2	3.0 ± 1.3	ns
A/L	753.9 ± 201.0	37.4 ± 21.2**	44.2 ± 16.0	0.0002
**Whole blood parameter**				
HbA1c [%]	3.483 ± 0.048	3.667 ± 0.033*	3.583 ± 0.075	0.0390

Obesity-related parameters were measured in dams from the control (CO; *n* = 4–6), obese (OB; *n* = 5–6), and obese with gestational metformin treatment (OB + M; *n* = 4–6) groups. Plasma parameters were analyzed using multiplex ELISA (Milliplex, Merck Millipore). Glycated hemoglobin (HbA1c) was freshly measured with a DCA Vantage analyzer (Siemens Healthineers) from whole blood samples. The adiponectin-to-leptin ratio (A/L) was calculated as described by Frühbeck et al. (2017) [41].

Asterisk (*) indicates differences between the OB and the CO groups. For MCP-1, four values (two from CO, one from OB, and one from OB + M groups) could not be measured due to low concentrations. Data are presented as mean ± SEM, Kruskal-Wallis + Dunn’s post-hoc test.

* *p* < 0.05, ** *p* < 0.01.

**Fig. 4:**
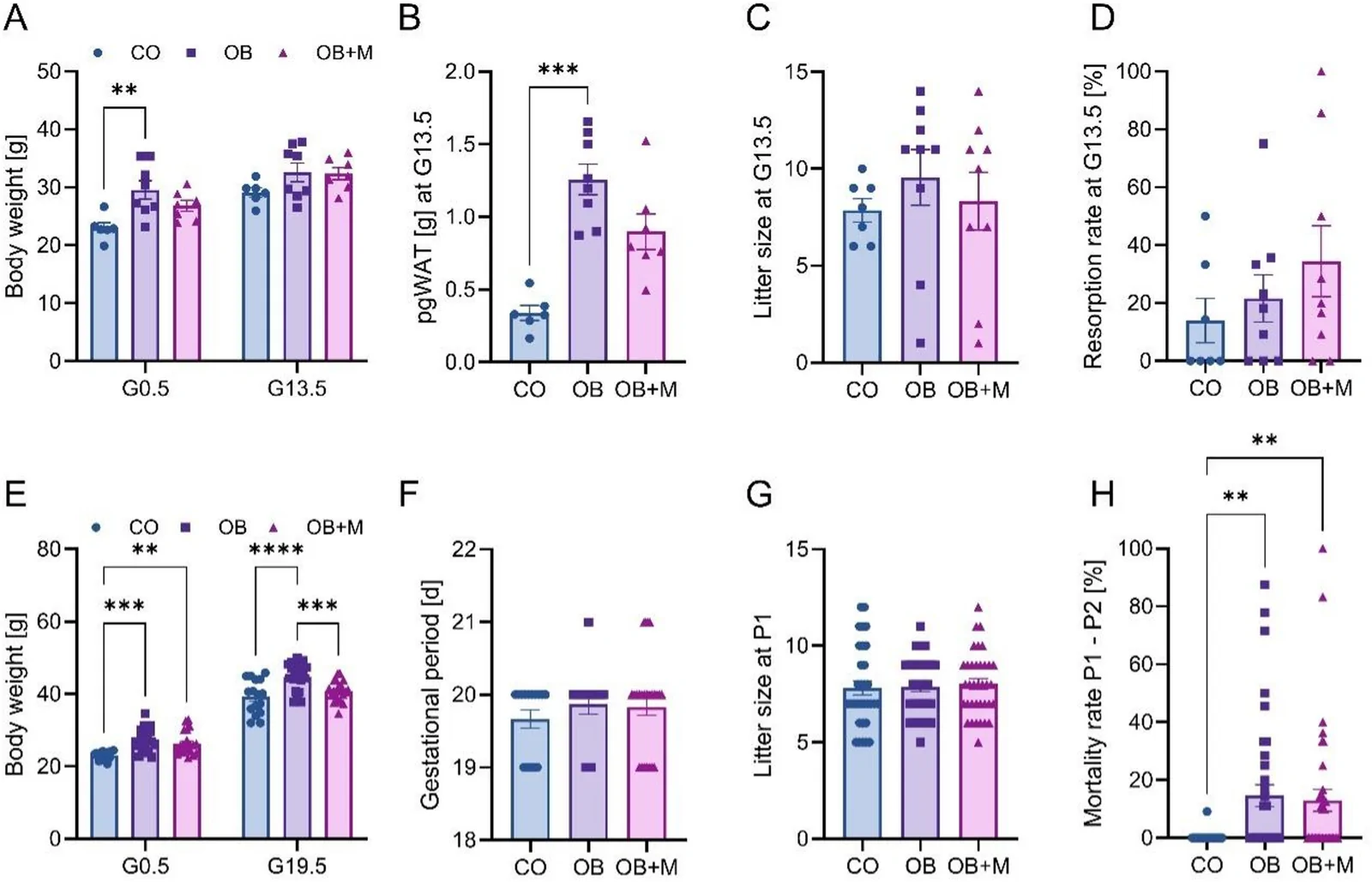
Pregnancy- and birth-related data of breeding dams and litters. (**A**) Body weight after mating and at G13.5 and (**B**) perigonadal white adipose tissue (pgWAT) weight of pregnant dams in control (CO; *n* = 6), obese (OB; *n* = 8), and obese with gestational metformin treatment (OB + M; *n* = 7) groups. (**C**) Litter size (live and resorbed fetuses) at G13.5 of CO (*n* = 9), OB (*n* = 7), and OB + M (*n* = 9) groups and (**D**) corresponding resorption rate. (**E**) Body weight after mating and at G19.5 of pregnant dams in CO (*n* = 16), OB (*n* = 21), and OB + M (*n* = 22) groups. (**F**) Gestation period of CO (*n* = 15), OB (*n* = 15), and OB + M (*n* = 30) dams. (**G**) Litter size at P1 of CO (*n* = 25), OB (*n* = 32), and OB + M (*n* = 30) litters, and (**H**) mortality rate (deceased pups from P1 to P2). Data are presented as mean ± SEM, two-way repeated-measurements ANOVA + Tukey’s post-hoc test (**A** and **E**) and Kruskal-Wallis + Dunn’s post-hoc test (**B**, **C**, **F**, and **H**) and one-way ANOVA + Tukey’s post-hoc test (**G**); * *p* < 0.05, ** *p* < 0.01, *** *p* < 0.001, **** *p* < 0.0001.

Body weight, perigonadal white adipose tissue (pgWAT), and fetuses were further examined in this sub-cohort at G13.5. All groups displayed similar body weights, although OB dams had higher pgWAT weight compared to CO dams (1.258 ± 0.106 vs. 0.339 ± 0.051 [g]; *p* = 0.0005; Fig. 4B), while OB + M dams did not differ from either group. Adiposity index (pgWAT/body weight) showed the same pattern, with a higher index for the OB dams compared to the CO dams, while OB + M dams did not differ from either group (Add. Figure 1D). Live fetuses were counted, and a resorption rate (deceased fetuses per litter) was calculated. There were no significant differences in litter size nor in the resorption rate. However, multiple regression analysis showed that resorption rate was significantly predicted by maternal body weight at G13.5 (*p* = 0.0031), indicating an inverse association between maternal weight and resorption rate, as a high number of resorbed fetuses’ results in lighter dams (Add. Table 2). A trend towards a positive association of the OB group (*p* = 0.0968) and pgWAT weight (*p* = 0.0576) with resorption rate was also observed, although these were not statistically significant. The mean metformin concentration in whole fetal lysates was 5.0 ± 1.8 µg/g (*n* = 5), suggesting that maternal metformin reached the fetuses by mid-gestation. Please note that the metformin concentrations in the dams, measured in plasma samples, are not directly comparable to the metformin concentrations in the fetuses, measured in whole-tissue lysates.

In another cohort at G19.5, shortly before delivery, body weight differences were clearly evident, with OB dams being heavier than CO dams (44.76 ± 0.807 vs. 39.24 ± 1.176 [g]; *p* < 0.0001) and OB + M dams (44.76 ± 0.807 vs. 40.75 ± 0.605 [g]; *p* = 0.0023), while the body weight of OB + M dams was similar to that of CO dams (Fig. 4E). Since some studies suggest that maternal obesity can prolong gestation, we compared gestation periods; but found no significant differences (Fig. 4F). Litter size at P1 did not differ between the groups (Fig. 4G). The number of live neonates at P2 was recorded, and a mortality rate was calculated based on the number of neonates at birth (P1). Neonates in the OB group exhibited an increased mortality rate compared to the CO group (14.62 ± 3.77 vs. 0.28 ± 0.284 [%]; *p* = 0.0010; Fig. 4H), which was not mitigated by maternal metformin treatment. The OB + M group also showed an elevated neonatal mortality rate relative to the CO group (12.97 ± 3.80 vs. 0.28 ± 0.284 [%]; *p* = 0.0010; Fig. 4H). However, neonatal mortality rate was not reliably predicted by group, maternal body weight at G19.5, or percentage weight gain during pregnancy (Add. Table 3).

### Maternal metformin treatment in obese dams reduced placental weight in a subgroup of female fetuses

**Fig. 5:**
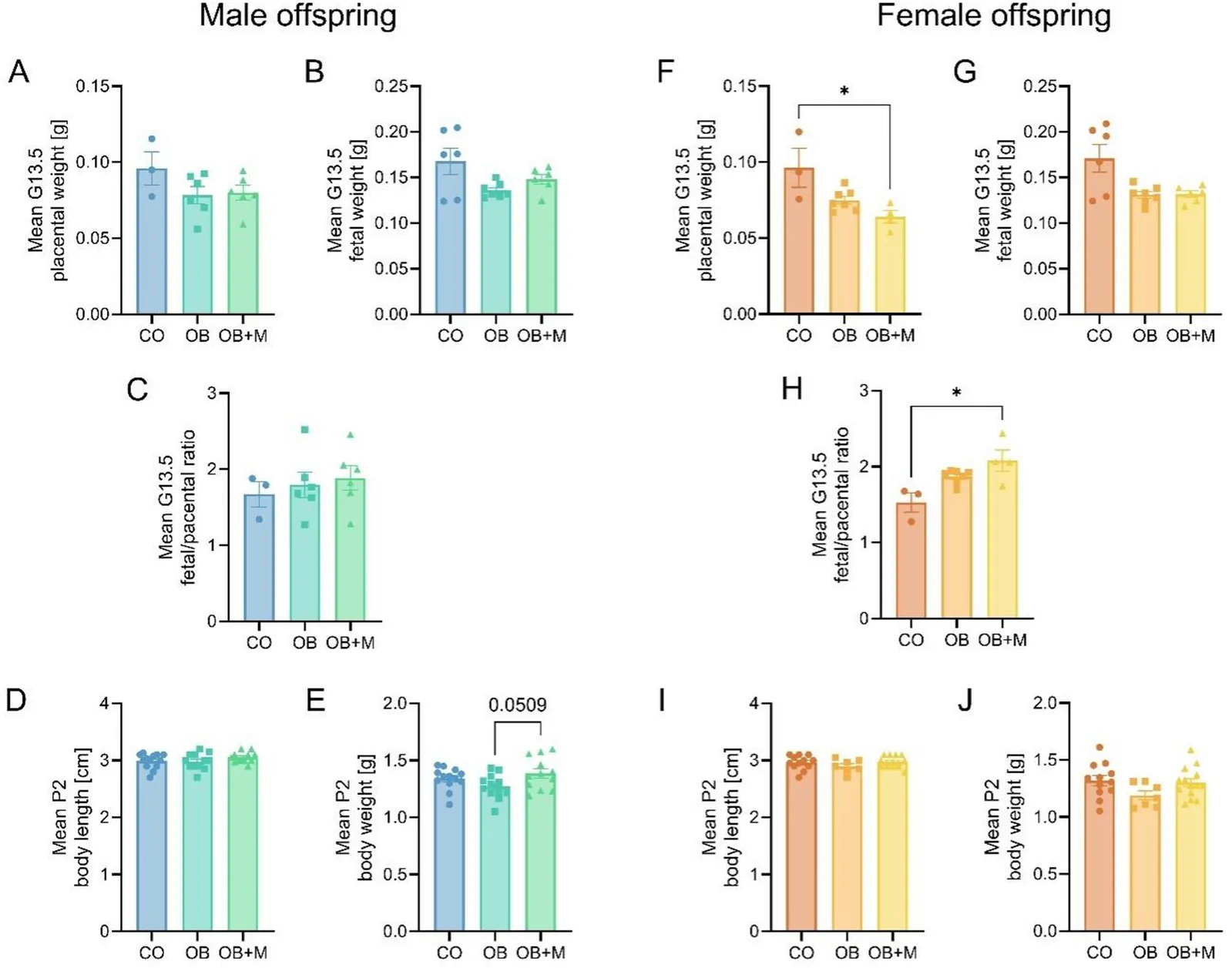
Fetal, placental, and neonatal phenotypes of male and female offspring at G13.5 and P2. (**A**) Mean weight of placentas, (**B**) mean weight of fetuses, and (**C**) mean fetal-to-placental weight ratio of male fetal-placental units per dam at G13.5 in control (CO; *n* = 3–6), obese (OB; *n* = 6–7), and obese with gestational metformin treatment (OB + M; *n* = 6) groups. (**D**) Mean body length and (**E**) mean body weight of male neonates at P2 in CO (*n* = 12), OB (*n* = 11), and OB + M (*n* = 11) groups. (**F**) Mean weight of placentas, (**G**) mean weight of fetuses, and (**H**) mean fetal-to-placental weight ratio of female fetal-placental units per dam at G13.5 in CO (*n* = 3–6), OB (*n* = 7), and OB + M (*n* = 4–6) groups. (**I**) Mean body length and (**J**) mean body weight of female neonates at P2 in CO (*n* = 12), OB (*n* = 7), and OB + M (*n* = 14) groups. Each sample represents the mean per litter. Data are presented as mean ± SEM, Kruskal-Wallis + Dunn’s post-hoc test (**A**-**C** and **F**-**J**) and one-way ANOVA + Tukey’s post-hoc test (**D **+ **E**); * *p* < 0.05.

Sex-dependent differences of placental and fetal weight were further investigated in the sub-cohort at G13.5. Regression and correlation analyses showed a significant positive relation of mean placental weight with litter size (Add. Figure 2A) in male OB fetuses and a significant negative relation of mean body weight and length with litter size in male OB + M neonates (Add. Figure 2E + F). Male fetuses showed no differences in mean placental or mean fetal weight at G13.5 (Fig. 5A + B). The ratio of fetal-to-placental weights was calculated as a measure of placental efficacy. Male fetuses showed no differences in their mean fetal-to-placental weight ratios (Fig. 5C). Female fetuses showed a reduced mean placental weight in the OB + M group compared to the CO group (0.0640 ± 0.0041 vs. 0.0963 ± 0.0129 [g]; *p* = 0.0234; Fig. 5F), while fetal weight did not differ (Fig. 5G). In female fetuses, the ratio in the OB + M group was elevated compared to the CO group (2.078 ± 0.1412 vs. 1.530 ± 0.1274; *p* = 0.0145; Fig. 5H), raising the possibility of altered placental supply dynamics. Neonatal outcomes at P2 showed no differences in mean body length or mean body weight in male (Fig. 5D + E) or female (Fig. 5I + J) offspring.

### Maternal metformin treatment in obese dams was associated with accelerated early postnatal weight gain and improved sensory-motor reflexes in neonatal offspring in both sexes

**Fig. 6:**
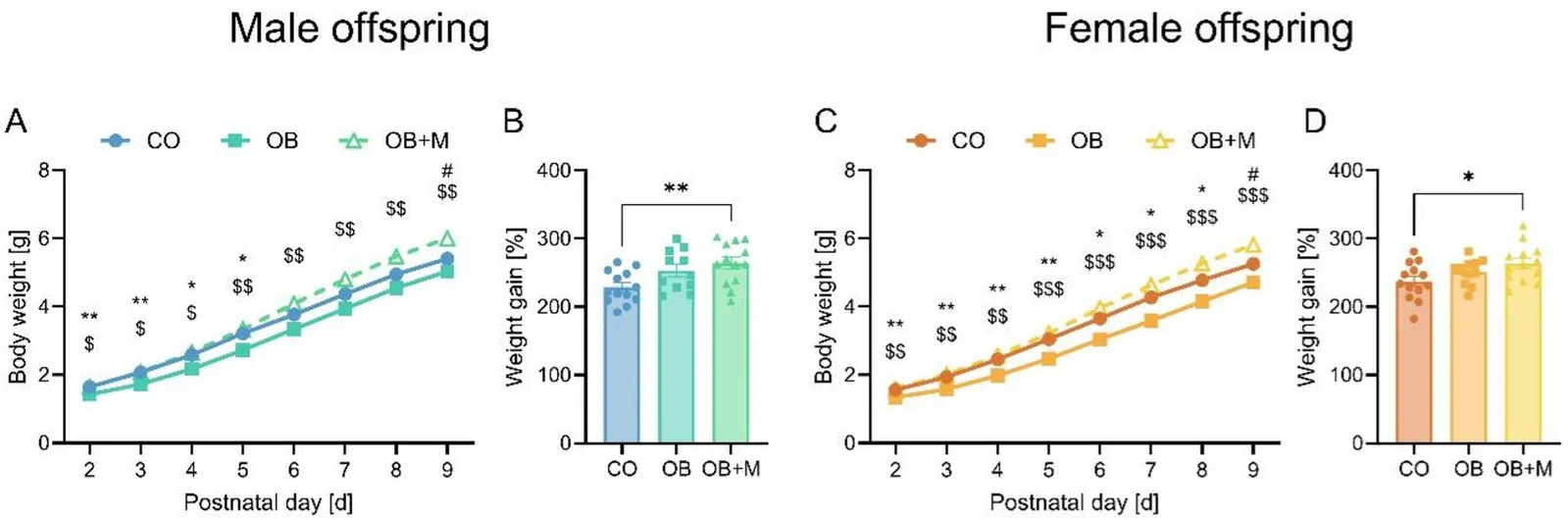
Early postnatal development of body weight in male and female neonatal offspring from P2-P9. (**A**) Body weight and (**B**) percentage body weight gain of male offspring, as well as (**C**) body weight and (**D**) percentage body weight gain in female offspring over the neonatal period of control (CO; *n* = 13), obese (OB; *n* = 11), and obese with gestational metformin treatment (OB + M; *n* = 13) groups. Asterisk (*) indicates differences between OB and CO, hash (^#^) indicates differences between OB + M and CO, and dollar (^$^) indicates differences between OB + M and OB (for line charts). Data are presented as mean ± SEM, two-way repeated-measurements ANOVA + Tukey’s post-hoc test (for line charts) and one-way ANOVA + Tukey’s post-hoc test (for bar charts); */^#^/^$^*p* < 0.05, **/^##^/^$$^*p* < 0.01, ***/^###^/^$$$^*p* < 0.001.

After delivery, early postnatal development was monitored from P2–P9 by daily weighing the neonatal offspring. From P2–P5, the body weight of male OB neonates differed from that of CO neonates, with OB neonates being lighter than CO neonates (Fig. 6A), while from P2–P9, male OB + M neonates differed from OB neonates, with OB + M neonates being heavier than OB neonates at every measured time point. At P9, the body weight of male OB + M neonates also exceeded that of CO neonates (6.004 ± 0.1786 vs. 5.408 ± 0.3050 [g]; *p* = 0.0366), resulting overall in an elevated weight gain of male OB + M neonates compared to CO neonates (264.2 ± 8.57 vs. 228.5 ± 6.56 [%]; *p* = 0.0073; Fig. 6B).

A similar pattern of early postnatal development was observed in the females, with OB neonates being lighter than CO neonates, from P2–P8, while OB + M neonates being heavier than OB neonates at every measured time point (Fig. 6C). At P9, the body weight of female OB + M neonates also exceeded that of CO neonates (5.810 ± 0.1568 vs. 5.588 ± 0.3184 [g]; *p* = 0.0454), with overall weight gain during the early postnatal period being the greatest for female OB + M neonates compared to CO neonates (263.6 ± 7.59 vs. 237.3 ± 7.55 [%]; *p* = 0.0300; Fig. 6D).

**Fig. 7:**
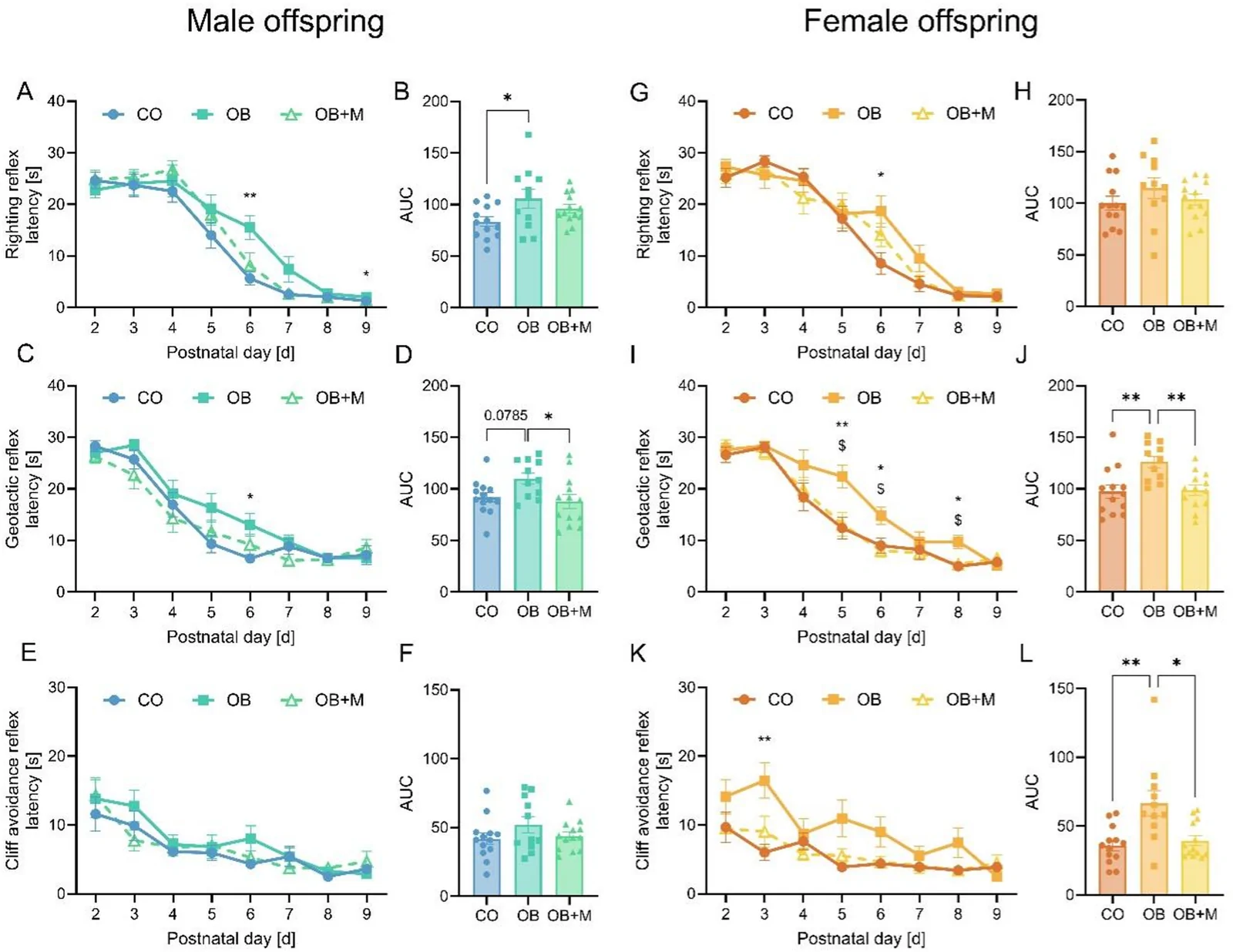
Early postnatal development of sensory-motor reflexes of male and female neonatal offspring from P2-P9. (**A**) Latency of righting reflex (RR) and (**B**) corresponding area under the curve (AUC), (**C**) latency of geotactic reflex (GR) and (**D**) corresponding AUC, (**E**) latency of cliff avoidance reflex (CA) and (**F**) corresponding AUC in the neonatal period of male control (CO; *n* = 13), obese (OB; *n* = 11), and obese with gestational metformin treatment (OB + M; *n* = 13) groups. (**G**) Latency of RR and (**H**) corresponding AUC, (**I**) latency of GR and (**J**) corresponding AUC, (**K**) latency of CA and (**L**) corresponding AUC in the neonatal period of female CO (*n* = 13), OB (*n* = 11), and OB + M (*n* = 13) groups. Asterisk (*) indicates differences between OB and CO, and dollar (^$^) indicates differences between OB + M and OB (for line charts). Data are presented as mean ± SEM, two-way repeated-measurements ANOVA + Tukey’s post-hoc test (for line charts) and one-way ANOVA + Tukey’s post-hoc test (for bar charts) or Kruskal-Wallis + Dunn’s post-hoc test (L); */^$^*p* < 0.05, **/^$$^*p* < 0.01.

Sensory-motor reflexes were tested daily from P2–P9 by assessing RR, GR, and CA reflexes. Male OB neonates showed significantly delayed latencies in the RR compared to CO neonates at P6 (15.50 ± 2.313 vs. 5.654 ± 1.236 [s]; *p* = 0.0049) as well as at P9 (2.045 ± 0.2073 vs. 1.269 ± 0.1076 [s]; *p* = 0.0120; Fig. 7A). The AUC was calculated and showed a higher value in the OB group compared to CO group (105.7 ± 9.3 vs. 83.45 ± 4.5; *p* = 0.0375; Fig. 7B). Maternal metformin treatment showed a slightly improved latency curve in male OB + M neonates, but the AUC did not differ significantly from the CO group. The GR was delayed in OB neonates compared to CO neonates at P6 (13.00 ± 2.247 vs. 6.50 ± 0.5746 [s]; *p* = 0.0412; Fig. 7C). The AUC showed a non-significant trend toward elevation in the OB group compared to the CO group (109.9 ± 5.4 vs. 91.69 ± 4.6; *p* = 0.0785), while the OB group showed a significantly higher AUC than the OB + M group (109.9 ± 5.4 vs. 87.76 ± 6.6; *p* = 0.0266; Fig. 7D). In the CA, latencies did not differ across tested time points in male offspring (Fig. 7E), and the AUC did not differ significantly between groups (Fig. 7F).

A similar pattern in sensory-motor reflex development was observed in the female neonates. The RR showed a significantly delayed latency in OB neonates compared to CO neonates at P6 (18.64 ± 3.069 vs. 8.577 ± 2.120 [s]; *p* = 0.0370; Fig. 7G). The AUC did not differ significantly between groups in the RR (Fig. 7H). The GR showed delayed latencies in OB neonates compared to CO neonates at P5 (22.41 ± 2.234 vs. 12.42 ± 2.082 [s]; *p* = 0.0096), at P6 (14.77 ± 1.691 vs. 9.00 ± 1.438 [s]; *p* = 0.0427) as well as at P8 (9.727 ± 1.285 vs. 5.00 ± 0.4634 [s]; *p* = 0.0114; Fig. 7I). The female OB neonates also demonstrated delayed latencies compared to OB + M neonates at P5 (22.41 ± 2.234 vs. 13.15 ± 2.328 [s]; *p* = 0.0233), at P6 (14.77 ± 1.691 vs. 8.038 ± 1.346 [s]; *p* = 0.0144) as well as at P8 (9.727 ± 1.285 vs. 5.385 ± 0.3497 [s]; *p* = 0.0182). The AUC differed in the OB group compared to the CO group (126.0 ± 5.5 vs. 97.37 ± 6.5; *p* = 0.0038) as well as to the OB + M group (126.0 ± 5.5 vs. 98.63 ± 4.9; *p* = 0.0057; Fig. 7J). In the CA, female OB neonates exhibited a delayed latency compared to CO neonates at P3 (16.45 ± 2.574 vs. 6.00 ± 1.166 [s]; *p* = 0.0063; Fig. 7K). The AUC showed higher values in the OB group compared to the CO group (66.46 ± 9.2vs. 35.98 ± 3.8; *p* = 0.007) as well as to the OB + M group (66.46 ± 9.2 vs. 39.48 ± 3.6; *p* = 0.0316; Fig. 7L).

To cross-validate the AUC-based findings, a complementary maturation-day analysis confirmed that OB offspring reached the criterion latency (≤ 5 s for RR and CA; ≤ 8 s for GR) consistently later than CO offspring across all three reflexes and both sexes, while OB + M offspring reached criterion at the same time as or earlier than CO offspring (Add. Table 4).

In summary, maternal metformin treatment was associated with accelerated early postnatal weight gain and improved sensory-motor reflex performances in both male and female OB + M neonates.

## Discussion

The present study investigated the effects of maternal metformin treatment during pregnancy in obese dams on maternal and fetal pregnancy-related outcomes, as well as the development of sensory-motor reflexes as an indicator of early CNS maturation in neonatal offspring. Potential benefits of metformin exposure were observed, adding to the existing knowledge of the short-term advantages of gestational metformin treatment during obese pregnancy.

Metformin-exposed OB + M neonates showed improved sensory-motor reflex maturation relative to OB neonates in both sexes, most consistently for the GR and CA in female neonates, and for the GR in male neonates, where AUC values of OB + M groups differed significantly from OB and no longer differed from CO. For the RR, the AUC values of male OB neonates were significantly elevated compared to male CO neonates, while OB + M males did not differ significantly from either group. AUC values of female OB + M neonates did not differ from either OB or CO groups. Sensory-motor reflexes such as RR, GR, and CA are well-established indicators of early neuromotor maturation. These reflexes depend on the integrity of vestibular, proprioceptive, and cerebellar networks that enable postural adjustment, spatial orientation, and coordinated motor execution during early development [21–23]. Male and female offspring of OB dams exhibited clear delays in reflex acquisition and prolonged response latencies, suggesting a detrimental impact of maternal obesity on early neurodevelopment. Our findings are consistent with other studies that investigated the influence of an HFD on neonatal reflex ontogeny [28–30]. Offspring exposed to maternal HFD at different stages of development, either during pregnancy [25], during lactation [28, 29], or combined [30], all found adverse effects on reflex ontogeny. One study found no adverse impact on offspring reflex ontogeny when investigating the effects of a WSD containing 14.5% fat, although offspring showed elevated somatic growth, with heavier neonates [45]. However, the WSD examined here was more similar to the SD’s fat content in the other studies mentioned. Mixed results have been reported on offspring postnatal somatic growth, particularly in studies examining exposure to higher lipid contents (~ 50%) at varying exposure times [28–30]. While some studies observed decreased somatic growth with smaller and lighter offspring and accompanied delayed maturation of physical features [28, 29], others found no differences in early somatic growth but showed accelerated development in neonatal offspring exposed to maternal HFD during pregnancy and lactation [30]. Reflexes reflect a complex network of CNS integration and communication between sensory input and motor output. It is essential to note that the testing methods employed here provide only a general correlation. The development of physical features and the ontogeny of reflex responses depend heavily on the lipid source, quantity, and timing of administration [40]. Our study examined a maternal diet comprising 45% fat, mainly derived from lard and soybean oil, with a high sucrose content. This diet led to maternal obesity in mice and adversely affected the offspring’s early development.

In line with the observed delay in sensory-motor reflex development, both male and female OB offspring displayed significantly reduced body weights (in OB males from P2–P5, in OB females from P2–P8), despite comparable percentage weight gain with CO offspring. Maternal obesity, although associated with nutrient excess, can paradoxically lead to fetal growth restriction (FGR) due to impaired placental function, as demonstrated in both clinical data and animal models [46–49]. At G13.5, only OB + M female fetuses exhibited significantly reduced placental weights and showed aberrant fetal-to-placental weight ratios, suggesting a change in placental growth dynamics. Maternal obesity in mouse models was associated with decreased fetal-to-placental weight ratios, suggesting reduced placental efficiency [50]. Our results, showing an increased fetal-to-placental ratio in OB + M, could indicate increased placental efficiency as a compensatory response to FGR. However, fetal body weights in both male and female OB and OB + M offspring were not statistically significantly reduced after litter size correction, due to considerably high variances in the CO groups and a relatively small sample size. Strikingly, reduced fetal and postnatal weight, as well as delayed reflexes in OB offspring, resemble findings from models of prenatal malnourishment [51, 52], further supporting an FGR-like phenotype that was not rescued by maternal metformin administration. Furthermore, a high body mass index during pregnancy has been associated with a more extended gestation period [53, 54], probably due to obesity-related hormonal and perinatal physiological changes that influence the timing of delivery [55, 56]. However, we found no evidence of prolonged gestation in the WSD-exposed groups in this project. This discrepancy may reflect differences in dietary composition between studies, but more likely relates to species differences, as the reported association derived from clinical cohorts, whereas murine gestational length is short and tightly regulated. Nonetheless, OB + M offspring showed accelerated early postnatal weight gain and improved reflex ontogeny, despite reduced placental weight of female fetuses at mid-gestation. While this may suggest a partial rescue through maternal metformin treatment, both FGR and rapid postnatal growth have been linked to an increased risk of obesity and metabolic disorders later in life [57, 58], underscoring the need for long-term monitoring of growth trajectories in metformin-exposed offspring.

Interestingly, although maternal metformin treatment accelerated neonatal growth and improved neurodevelopmental markers, it did not prevent FGR and was associated with decreased placental weight in female fetuses at mid-gestation. This suggests that early gestational metformin administration (from G0.5 onward) may be insufficient to fully normalize the intrauterine environment. One potential explanation is restricted placental transfer of metformin during early gestation. The placental key transporter, organic cation transporter 3 (OCT3), which mediates maternal-to-fetal metformin transfer, shows markedly increased expression in late gestation (up to 128-fold increase between G10 and G19) [59, 60], suggesting that fetal metformin exposure increases substantially toward term. This implies that the G13.5 timepoint assessed here may represent a relatively early window of fetal metformin exposure, and that more pronounced effects on placental adaptation and fetal programming might be expected at later gestational timepoints. The metformin concentration in whole fetal lysates at G13.5 with 5 µg/g was broadly comparable to organ-specific concentrations reported in murine fetal kidney (24.5 µg/g) or fetal liver (2.3 µg/g) lysates at G18.5 in a study by Hufnagel et al. (2022), who investigated fetoplacental health of obese dams after metformin exposure (please note that Hufnagel et al., (2022) provided the concentration in nmol/g; for better comparability, we converted the values to µg/g using the molecular weight of metformin base, 129.16 g/mol), though direct comparison is limited by the difference between whole-body lysates and organ-specific measurements. However, it is essential to note that despite improved metabolic profiles of pregnant dams, no beneficial effects of maternal metformin treatment have been observed on fetal growth at mid-gestation (G15.5) or even at late gestation (G18.5) [61, 62].

Importantly, OB + M dams continued to receive the WSD throughout lactation (identical to OB dams), meaning that the accelerated postnatal weight gain in OB + M offspring cannot be attributed to a direct postnatal effect of metformin, which was discontinued at birth, nor to differences in dietary fat exposure via maternal milk between OB and OB + M dams. Alternatively, postnatal factors such as improved maternal care, altered milk composition, or shifts in maternal or neonatal metabolism might have driven the observed acceleration of postnatal growth. Maternal HFD before and during pregnancy has been shown to act as a stressor, increasing maternal glucocorticoid signaling to the fetus and disrupting maternal care behavior toward the neonatal offspring [63], potentially through impaired olfactory processing and pup recognition. The increased neonatal mortality observed in OB + M animals in our study suggests that metformin treatment does not benefit these behavioral impairments. Future studies should directly assess the impact of metformin on maternal caregiving behavior and olfactory function. Furthermore, metformin exposure during lactation may have additional effects. In one study, maternal metformin administration during lactation reduced milk amino acid content and significantly altered mammary gland gene expression, resulting in smaller pups [64]. It remains uncertain how discontinuation of metformin after birth would have altered milk composition and whether and how sequential gestational and lactational exposure to metformin might have influenced neonatal development.

Consistent with previous findings, our data show that metformin treatment significantly attenuated gestational weight gain compared with untreated OB dams, with OB + M dams no longer differing significantly from CO by the end of gestation. In contrast, adiposity, circulating leptin levels, and HbA1c in OB + M dams showed only a numerical trend toward lower values relative to OB dams, without reaching statistical significance, likely reflecting the limited sample size of the mid-gestation cohort. The adiponectin-to-leptin ratio, however, remained similar to the OB group and did not show a comparable trend, indicating that metformin’s effects on maternal metabolic parameters were not uniform across all measures. The attenuated weight gain and the numerical trends in adiposity and leptin values are consistent with clinical trials reporting metformin’s effectiveness in reducing maternal weight gain and improving metabolic parameters during gestation, particularly in women with GDM [32, 65, 66] and in obese women [33, 34, 67, 68]. Leptin is a key adipokine implicated in placental development and fetal metabolic programming [69], and elevated maternal leptin levels, as well as leptin resistance, have been associated with adverse neurodevelopmental and metabolic trajectories in offspring [70]. Given the trend toward reduced gestational weight gain, adiposity, and leptin levels in OB + M dams, a partial modulation of the maternal metabolic and endocrine environment is biologically plausible and may represent one pathway through which maternal treatment could have influenced offspring development [43]. However, this remains speculative given the lack of statistical significance for individual parameters and the absence of direct mediation analysis. The mid-gestation cohort was relatively small, and statistical testing was correspondingly underpowered, which limits the strength of any firm conclusion. Nevertheless, the direction and consistency of the observed changes align with the expected physiological actions of metformin and highlight biologically plausible pathways warranting further investigation.

Interestingly, while metformin-treated dams showed improvements in weight-related parameters and a tendency toward normalization of fasted blood glucose and HbA1c levels, their glucose tolerance did not improve relative to OB dams. In fact, glucose levels in OB + M dams during ipGTT were numerically higher than in untreated OB dams, without reaching statistical significance, a finding also observed in another study in our group using maternal HFD [61]. This paradoxical response contrasts with metformin’s classical role in enhancing insulin sensitivity and lowering hepatic glucose production [71]. According to a review, 21–46% of pregnant women with GDM treated with metformin were administered additional insulin to reach target glucose levels [35]. Obesity, age, and early-onset metformin treatment were identified as factors associated with an increased need for additional insulin in GDM therapy [72, 73].

The metformin concentration in our study reached approximately 3 µg/mL in maternal plasma at mid-gestation. This concentration was twice as high as measured in obese mice treated with 300 mg/kg/d metformin [62] and in the plasma of women treated for GDM in late pregnancy [36, 74]. Doses ranged from 500 to 2500 mg/d of metformin, with 2000 mg/d as the standard dose for the treatment of GDM [75]. We have targeted a dose of 380 mg/kg/d, which is equivalent to a human dose of 30.78 mg/kg/d (~ 2000 mg/d for a 65 kg person) according to [76]. Dose application via drinking water was estimated based on the pre-gestational mean water intake in WSD-fed female mice. Water intake increased during pregnancy, leading to a higher metformin intake in the second half of gestation, when body weight, feed, and water intake naturally rise to sustain pregnancy (Add. Figure 1A + B). As a result, the actual dose of metformin in our study was slightly higher (433.2 ± 59.96 mg/kg/d) than intended, though still ranging within common treatment doses (~ 2280 mg/d for a 65 kg person).

After oral administration, metformin is absorbed in the intestine and distributed to multiple organs, including the placenta [77, 78], via various membrane transporters. At the molecular level, metformin acts via both adenosine monophosphate-activated protein kinase (AMPK)-dependent and -independent pathways involving mitochondrial and lysosomal mechanisms [79–81]. Beyond its classical hepatic action, namely inhibition of gluconeogenic and glycogenolytic pathways [71], metformin exerts pleiotropic effects across multiple organ systems, including anti-inflammatory, anti-oxidative, anti-aging, and anti-proliferative actions [82]. These broad effects make metformin an attractive agent for improving poor glycemic control in pregnancies complicated by T2DM, GDM, obesity, or before pregnancy in polyendocrine metabolic ovarian syndrome (PMOS) [83, 84]. For example, the weight-reducing effect observed in our dams may reflect metformin’s cumulative influence on the gut-brain axis by modulating hypothalamic circuits that suppress appetite and enhance satiety, favorably alter the gut microbiota, and increase the release of cytokines and peptides that regulate energy intake and expenditure [85]. Moreover, metformin was found to reduce the teratogenic risks of neural tube and cardiovascular defects linked to poor glycemic control in diabetic pregnancies. However, some follow-up studies have associated *in utero* exposure with increased childhood obesity [77]. Still, the direct effects of metformin on placental structure and function remain under closer investigation. Metformin treatment in diabetic and obese mothers and HFD-fed mouse models stimulated epigenetic regulation of placental mitochondrial biogenesis and improved offspring glucose homeostasis [78], while in primary trophoblast cultures, markers of mitochondrial respiration, adenosine triphosphate (ATP) production, and oxidative stress were reduced [79]. Conversely, in our group’s study, metformin treatment improved endothelial cell markers, suggesting enhanced placental angiogenesis, but did not improve placental lipid deposition, peroxidation, calcification, or fetal vessel structures [61]. Nonetheless, the systemic impact of these mechanisms on long-term outcomes during fetal development remains context-dependent.

Of particular relevance to the observed normalization in neonatal sensory-motor reflexes is the emerging evidence for metformin’s direct neuroprotective effects on the developing brain. Maternal obesity has been shown to impair white matter development in the offspring brain, including reduced expression of myelin basic protein in fetal brain tissue, as well as impaired hippocampal myelination and reduced cortical thickness in adult offspring [86, 87]. Since the myelination of vestibular, cerebellar, and corticospinal tracts is critical for the maturation of sensory-motor reflexes in the neonatal period, obesity-induced disruption of this process might be responsible for the delayed reflex ontogeny observed in OB offspring. Metformin may counteract these effects through several complementary mechanisms. Via AMPK activation (metformin’s primary molecular target [79]), metformin supports oligodendrocyte differentiation and myelination [88]. Furthermore, metformin has been shown to attenuate white matter injury in neonatal mice, likely by reducing neuroinflammation and oxidative stress, thereby increasing the myelin basic protein expression and improving myelin sheath thickness [89]. Taken together, these findings suggest that maternal metformin treatment may have partially restored myelination and reduced neuroinflammation in the developing sensory-motor circuits of OB + M offspring, thereby facilitating more advanced reflex maturation.

In summary, the mechanisms through which maternal metformin treatment may have normalized early sensory-motor reflex development in OB + M offspring are probably multifactorial. First, metformin attenuated some obesity-related traits in the dams, indicating an overall shift towards a more favorable metabolic and endocrine maternal environment. Second, emerging evidence suggests that metformin’s positive effects on placental structure and function can improve and stabilize the intrauterine environment. Third, metformin readily crosses the placenta and reaches the developing fetus, where it may promote neurogenesis, decrease oxidative stress, and reduce neuroinflammation in the developing brain, supported by increasing evidence of metformin’s neuroprotective properties [90]. Although we did not directly assess CNS endpoints, it is plausible that both indirect effects on the maternal-placental metabolic environment and direct actions within the fetus jointly facilitated more advanced neuromotor maturation in OB + M neonates. However, due to the small mid-gestation sample size and the absence of direct CNS measures, these mechanistic links remain speculative and require confirmation through more targeted research and a larger cohort of dams and neonates.

## Conclusions

Taken together, these findings demonstrate that metformin did not restore maternal glucose tolerance during pregnancy in obese dams but significantly attenuated gestational weight gain and showed a numerical trend toward reduced white adipose tissue accumulation and plasma leptin levels, without reaching statistical significance for the latter two. These data suggest that metformin may act as a modulator of the maternal metabolic state beyond its glycemic control effects. Furthermore, maternal metformin treatment was associated with accelerated early postnatal growth and improved sensory-motor reflex maturation in OB + M neonates, most consistently for the GR in both sexes and CA reflexes in female neonates. Because metformin was discontinued after delivery while OB + M dams remained on the WSD throughout lactation, these effects reflect prenatal programming rather than ongoing pharmacological action. Overall, these data suggest that metformin holds potential to mitigate some adverse aspects of early postnatal developmental programming in offspring of obese mothers. However, to assess the long-term effects of accelerated early postnatal growth in OB + M neonates, including cognitive and metabolic assessments, long-term follow-ups into adolescence and adulthood will be necessary to determine whether maternal metformin treatment provides a safe and sustainable intervention for improving offspring health outcomes in the long run.

## Supplementary Information

Supplementary Material 1.

## Data Availability

The datasets used and/or analyzed during the current study are available from the corresponding author on reasonable request.

## Abbreviations

A/L: Adiponectin-to-leptin ratio
ACN: Acetonitrile
AMPK: Adenosine monophosphate-activated protein kinase
ATP: Adenosine triphosphate
AUC: Area under the curve
CNS: Central nervous system
CO: Control
DIO: Diet-induced obesity
DOHaD: Developmental Origins of Health and Disease
ELISA: Enzyme-linked immunosorbent assay
FGR: Fetal growth restriction
FIWI: Feed and water intake test
G: Gestational day
GDM: Gestational diabetes mellitus
HbA1c: Glycated hemoglobin
HClO_4_: Perchloric acid
HFD: High-fat diet
IL-6: Interleukin 6
ipGTT: Intraperitoneal glucose tolerance test
LC-MS: liquid chromatography-mass spectrometry
MCP-1: Monocyte chemoattractant protein 1
NRT: Neonatal reflex tests
OB: Obese
OB + M: Obese with metformin treatment
OCT3: Organic cation transporter 3
P: Postnatal day
PMOS: Polyendocrine metabolic ovarian syndrome
PCR: Polymerase chain reaction
pgWAT: Perigonadal white adipose tissue
SD: Standard diet
T2DM: Type 2 diabetes mellitus
TNFα: Tumor necrosis factor alpha
WSD: Western-style diet
