# Supplementary material for: Maternal metformin treatment in obese pregnant dams normalizes neonatal sensory-motor reflexes
Source: Mol Cell Pediatr. 2026 Sep 17;13:47. doi: 10.1186/s40348-026-00259-8 (PMC13582716; doi:10.1186/s40348-026-00259-8)
Supplement: Supplementary file 1 [file 40348_2026_259_MOESM1_ESM.docx]

**Additional File:**

Maternal Metformin Treatment in Obese Pregnant Dams Normalizes Neonatal Sensory-Motor Reflexes

**Elena Müller-Limberger^1^, Bettina Frederick^1^, Sebastian Hansen^1^, Maria Wohlfarth^1^, Ruth Janoschek^1^, Esther Mahabir^2^, Pascal Fischer^2^, Dirk Gründemann^3^, Simone Kalis^3^, Miguel A. Alejandre Alcázar^1,4,5^, Jörg Dötsch^1^, Eva Hucklenbruch-Rother^1^, and Sarah Appel^1*^**

^1^Department of Pediatrics and Adolescent Medicine, Faculty of Medicine and University Hospital Cologne, University of Cologne, Cologne, Germany.

^2^Comparative Medicine, Center for Molecular Medicine Cologne (CMMC), Faculty of Medicine and University Hospital Cologne, University of Cologne, Cologne, Germany.

^3^Department of Pharmacology, Faculty of Medicine and University Hospital Cologne, University of Cologne, Cologne, Germany.

^4^Cologne Excellence Cluster on Cellular Stress Responses in Aging-Associated Diseases (CECAD), Faculty of Medicine and University Hospital Cologne, University of Cologne, Cologne, Germany.

^5^Institute for Lung Health (ILH), University of Giessen and Marburg Lung Center (UGMLC) and Cardiopulmonary Institute (CPI), Member of the German Center for Lung Research (DZL).

***Correspondence:**
Dr. rer. nat. Sarah Appel (sarah.appel@uk-koeln.de)

# Additional Tables

***Additional Table 1: Dietary composition of chows used in the study.***

|  | **Standard diet (SD)** | **Western-style diet (WSD)** |
| --- | --- | --- |
| Company | Ssniff | Ssniff |
| Order number | V1534-000  (R/M Maintenance) | E15744-344  (Diet-induced obesity (DIO)) |
| **Energy [x/kg]** | | |
| Metab. energy [kcal] | 3225 | 4615 |
| Metab. energy [MJ] | 13.5 | 19.3 |
| Gross energy [MJ] | 16.2 | 22.5 |
| **Rough composition [kJ%]** | | |
| Fat | 9.0 | 45.0 |
| Protein | 24.0 | 20.0 |
| Carbohydrates | 67.0 | 35.0 |
| **Crude ingredients [%]** | | |
| Protein (N x 6.25) | 19.0 | 22.0 |
| Fat | 3.3 | 23.6 |
| Fiber | 5.0 | 5.7 |
| Ash | 6.4 | 5.3 |
| Starch | 35.9 | 6.8 |
| Sugar sucrose | 5.4 | 21.1 |
| N free extracts | 54.6 | 40.0 |
| **Minerals [%]** | | |
| Calcium | 1.00 | 0.92 |
| Phosphorus | 0.70 | 0.64 |
| Ca/P | 1.43:1 | 1.44:1 |
| Sodium | 0.24 | 0.20 |
| Magnesium | 0.22 | 0.23 |
| Potassium | 0.92 | 0.97 |
| **Fatty acids [%]** | | |
| C 12:0 | - | 0.05 |
| C 14:0 | 0.01 | 0.29 |
| C 16:0 | 0.45 | 5.33 |
| C 18:0 | 0.09 | 2.92 |
| C 20:0 | 0.01 | 0.07 |
| C 16:1 | 0.01 | 0.62 |
| C 18:1 | 0.62 | 9.42 |
| C 18:2 | 1.76 | 3.46 |
| C 18:3 | 0.23 | 0.37 |
| **Amino acids [%]** | | |
| Lysine | 1.00 | 1.83 |
| Methionine (Met) | 0.33 | 0.78 |
| Cystine (Cys) | 0.35 | 0.39 |
| Met + Cys | 0.68 | 1.18 |
| Threonine | 0.71 | 0.97 |
| Tryptophan | 0.25 | 0.29 |
| Arginine | 1.19 | 0.86 |
| Histidine | 0.48 | 0.67 |
| Valine | 0.90 | 1.54 |
| Isoleucine | 0.79 | 1.25 |
| Leucine | 1.39 | 2.19 |
| Phenylalanine | 0.88 | 1.14 |
| Phe + Tyr | 1.49 | 2.31 |
| Glycine | 0.88 | 0.47 |
| Glutamic acid | 4.10 | 4.97 |
| Aspartic acid | 1.79 | 1.64 |
| Proline | 1.29 | 2.53 |
| Serine | 0.99 | 1.32 |
| Alanine | 0.82 | 0.66 |
| **Vitamins [x/kg]** | | |
| Vitamin A [IU] | 15000 | 15000 |
| Vitamin D_3_ [IU] | 1100 | 1500 |
| Vitamin E [mg] | 110 | 150 |
| Vitamin K [mg] | 7 | 20 |
| Thiamine (B_1_) [mg] | 18 | 25 |
| Riboflavin (B_2_) [mg] | 22 | 16 |
| Pyridoxine (B_6_) [mg] | 21 | 16 |
| Cobalamin (B_12_) [µg] | 100 | 30 |
| Nicotinic acid [mg] | 115 | 47 |
| Pantothenic acid [mg] | 40 | 55 |
| Folic acid [mg] | 7 | 16 |
| Biotin [µg] | 510 | 300 |
| Choline [mg] | 1370 | 920 |
| **Trace elements [mg/kg]** | | |
| Iron | 189 | 168 |
| Manganese | 68 | 95 |
| Zinc | 91 | 65 |
| Copper | 15 | 13 |
| Iodine | 2.1 | 1.2 |
| Selenium | 0.3 | 0.2 |

Table adapted from ssniff manufacturer of special diets for laboratory rodents^[1]^.

***Additional Table 2: Multiple linear regression of pregnancy-related parameters at G13.5.***

| **Variable** | **VIF** | **Estimate** | **Standard error** | **95% CI** | **\|t\|** | ***p* value** | ***p* value**  **summary** |
| --- | --- | --- | --- | --- | --- | --- | --- |
| Group  [OB] | 4.670 | 25.50 | 14.40 | -5.18 to  56.18 | 1.771 | 0.0968 | ns |
| Group  [OB+M] | 2.581 | 11.63 | 11.44 | -12.76 to  36.01 | 1.016 | 0.3257 | ns |
| **Maternal**  **weight G13.5** | **3.405** | **-6.54** | **1.86** | **-10.50 to -2.57** | **3.513** | **0.0031** | ****** |
| Maternal  pgWAT weight | 4.722 | 31.72 | 15.43 | -1.16 to  64.60 | 2.056 | 0.0576 | ns |

Multiple linear regression was performed to determine whether the variables predict fetal resorption rate at G13.5. G: gestational day; ns: not significant; OB: obese group; OB+M: obese with gestational metformin treatment group; |t|: absolute t value of the coefficient test; VIF: variance inflation factor; ** p < 0.01.

***Additional Table 3: Multiple linear regression of birth-related parameters.***

| **Variable** | **VIF** | **Estimate** | **Standard error** | **95% CI** | **\|t\|** | ***p* value** | ***p* value**  **summary** |
| --- | --- | --- | --- | --- | --- | --- | --- |
| Group  [OB] | 2.365 | 15.69 | 10.41 | -5.33 to  36.72 | 1.507 | 0.1394 | ns |
| Group  [OB+M] | 2.074 | 12.73 | 9.28 | -6.01 to  31.48 | 1.372 | 0.1776 | ns |
| Maternal weight G19 | 1.608 | 1.02 | 1.16 | -1.32 to  3.36 | 0.881 | 0.3833 | ns |
| Maternal weight gain | 1.443 | -0.06 | 0.23 | -0.51 to  0.39 | 0.263 | 0.7941 | ns |

Multiple linear regression was performed to determine whether the variables predict neonatal mortality after delivery. G: gestational day; ns: not significant; OB: obese group; OB+M: obese with gestational metformin treatment group; |t|: absolute t value of the coefficient test; VIF: variance inflation factor

***Additional Table 4: Complementary maturation-day analysis for neonatal reflex tests.***

| **Reflex** | **Sex** | | **Group** | | **P2** | | **P3** | | **P4** | | **P5** | | **P6** | | **P7** | | **P8** | | **P9** | | **Maturation day (first median ≤ x s)** | |
| --- | --- | --- | --- | --- | --- | --- | --- | --- | --- | --- | --- | --- | --- | --- | --- | --- | --- | --- | --- | --- | --- | --- |
| **Righting reflex (RR)**  (first median ≤ 5 s) | |  | | CO | | 26 | | 30 | | 22 | | 13 | | **3,5** | | 3 | | 2 | | 1 | | P6 |
|  |  | Male | | OB | | 22 | | 30 | | 30 | | 17 | | 16,5 | | **4,5** | | 2,5 | | 2 | | P7 |
|  |  |  | | OB+M | | 27 | | 29,5 | | 30 | | 17 | | **3,5** | | 2,5 | | 1,5 | | 1,5 | | P6 |
|  |  |  | | CO | | 30 | | 30 | | 29,5 | | 18,5 | | **4,2** | | 2,5 | | 2 | | 2 | | P6 |
|  |  | Female | | OB | | 30 | | 30 | | 30 | | 17,5 | | 18,5 | | 5,5 | | **3** | | 2,5 | | P8 |
|  | |  | | OB+M | | 29 | | 30 | | 30 | | 21,5 | | 16 | | **3** | | 2,5 | | 2 | | P7 |
| **Geotactic reflex (GR)**  (first median ≤ 8 s) | |  | | CO | | 30 | | 30 | | 13 | | **7** | | 6 | | 6,5 | | 6,5 | | 4,5 | | P5 |
|  |  | Male | | OB | | 30 | | 30 | | 19,5 | | 18,5 | | 11 | | **8** | | 5,5 | | 6,5 | | P7 |
|  |  |  | | OB+M | | 30 | | 30 | | 8 | | 8,5 | | **7** | | 5,5 | | 6,5 | | 7,5 | | P6 |
|  |  |  | | CO | | 30 | | 30 | | 19 | | 10 | | **7** | | 6 | | 4,5 | | 5,5 | | P6 |
|  |  | Female | | OB | | 30 | | 30 | | 30 | | 20,5 | | 17,5 | | **7** | | 8,5 | | 4,5 | | P7 |
|  | |  | | OB+M | | 30 | | 30 | | 19,5 | | 8,5 | | **7** | | 7 | | 5,5 | | 5,5 | | P6 |
| **Cliff avoidance (CA)**  (first median ≤ 5 s) | |  | | CO | | 8,5 | | 7,5 | | 7,5 | | **5** | | 4 | | 3 | | 2 | | 3 | | P5 |
|  |  | Male | | OB | | 13 | | 10 | | 6,5 | | **4,5** | | 4,5 | | 4 | | 3 | | 2,5 | | P5 |
|  |  |  | | OB+M | | 12 | | 5,5 | | **5** | | 5 | | 5 | | 3 | | 2,5 | | 3 | | P4 |
|  |  |  | | CO | | 6 | | **4,5** | | 9,5 | | 3,5 | | 4 | | 2,5 | | 2,5 | | 3,5 | | P3 |
|  |  | Female | | OB | | 17 | | 17,5 | | 7,5 | | 7 | | 6,5 | | **4,5** | | 3 | | 2,5 | | P7 |
|  | |  | | OB+M | | 7,5 | | 5,5 | | **5** | | 5 | | 3 | | 3 | | 2,5 | | 3 | | P4 |

Maturation day: first postnatal day on which group median latency ≤ 5 s (RR, CA) or < 8 s (GR) within P2–P9.

# Additional Figures

***Additional Figure 1: Metformin intake and percentage weight gain during pregnancy (G0-19) and adiposity index at G13.5.*** *(A) Metformin intake curve and (B) corresponding mean dosage in the obese with gestational metformin treatment (OB+M; n = 11) group. (C) Percentage weight gain throughout pregnancy of control (CO; n = 16), obese (OB; n = 21), and OB+M (n = 22) dams. (D) Adiposity index (percentage of pgWAT weight [g]/body weight [g]) for dams at G13.5 of CO (n = 6), OB (n = 8), and OB+M (n = 7). Data are presented as mean ± SEM, unpaired Student’s t-test (B), one-way ANOVA + Tukey’s post-hoc test (C) and Kruskal-Wallis test + Dunn’s post-hoc test (D); * p < 0.05, *** p < 0.001.*

***Additional Figure 2: Regression and correlation analysis of phenotypic data with litter size of fetal and neonatal male and female offspring.*** *(A) Correlation of mean placental weight with litter size of male fetuses in control (CO; n = 3), obese (OB; n = 6), and obese with gestational metformin treatment (OB+M; n = 6) groups. (B) Correlation of mean fetal weight with litter size in male fetuses in CO (n = 6), OB (n = 7), and OB+M (n = 6) groups. (C) Correlation of mean placental weight with litter size of female fetuses in CO (n = 3), OB (n = 7), and OB+M (n = 4) groups. (D) Correlation of mean fetal weight with litter size in female fetuses in CO (n = 6), OB (n = 7), and OB+M (n = 6) groups. (E) Correlation of mean neonatal length with litter size of male offspring in CO (n = 12), OB (n = 11), and OB+M (n = 11) groups. (F) Correlation of mean neonatal weight with litter size of male offspring in CO (n = 12), OB (n = 11), and OB+M (n = 11) groups. (G) Correlation of mean neonatal length with litter size of female offspring in CO (n = 12), OB (n = 7), and OB+M (n = 14) groups. (F) Correlation of mean neonatal weight with litter size of female offspring in CO (n = 12), OB (n = 7), and OB+M (n = 14) groups. Each sample represents the mean per litter. Simple linear regression with two-tailed Spearman correlation. Regression coefficients (r) and p values are indicated in the figures.*

1. Ssniff manufacturer of special diets for laboratory rodents. [https://www.ssniff.de/index.php?pcid=9&pdid=15.](https://www.ssniff.de/index.php?pcid=9&pdid=15) Accessed on 31 October 2025.
